# Male accessory breast carcinoma: a rare case report and literature review

**DOI:** 10.3389/fonc.2025.1635515

**Published:** 2025-09-05

**Authors:** Cheng Luo, Songli Wu, Ying Zeng, Song Zhao

**Affiliations:** 1 Department of General Surgery, The Thirteenth People’s Hospital of Chongqing (Chongqing Geriatrics Hospital), Chongqing, China; 2 Department of Pathology, The Thirteenth People’s Hospital of Chongqing (Chongqing Geriatrics Hospital), Chongqing, China

**Keywords:** accessory breast carcinoma, male breast cancer, axillary mass, TRPS1, immunohistochemistry

## Abstract

**Background:**

Accessory breast carcinoma, arising from embryologically persistent mammary tissue, is exceptionally rare in males, accounting for 2.4%-5.3% of all accessory breast malignancies. Due to limited clinical data, standardized diagnostic and therapeutic protocols remain undefined.

**Case presentation:**

A 72-year-old male presented with a 3-year history of a painless, mobile right axillary mass. Ultrasonography revealed a solid hypoechoic nodule with moderate vascularity. Histopathological examination confirmed invasive ductal carcinoma (grade II) with infiltrative growth, ER/PR/AR positivity (90%), and TRPS1 expression, confirming mammary origin. Adjuvant therapies were declined by the patient.

**Discussion:**

Differential diagnoses for axillary masses include fibroadenoma, lymphadenopathy, and cutaneous adnexal tumors. Immunohistochemistry (TRPS1, GATA-3) and histomorphology (absence of apocrine differentiation) are pivotal for distinguishing accessory breast carcinoma from mimics. Current management aligns with male breast cancer guidelines, emphasizing surgical resection, lymph node dissection, and adjuvant therapies tailored to molecular profiles. Over 90% of male breast cancers are hormone receptor-positive, warranting endocrine therapy.

**Conclusion:**

Male accessory breast carcinoma poses significant diagnostic challenges due to its rarity and nonspecific presentation. Clinicians should consider this entity in differential diagnoses of axillary or inguinal masses, irrespective of patient sex. Core needle biopsy and advanced imaging aid preoperative evaluation. Multimodal treatment, including surgery and risk-stratified adjuvant therapies, is critical for optimizing outcomes. This case underscores the need for heightened clinical suspicion and evidence-based guidelines to address this understudied malignancy.

## 1 Introduction

Accessory breast tissue originates from embryonic mammary ridges extending from the axillary to inguinal regions, resulting from incomplete regression during embryogenesis. Malignant transformation occurs in the epithelial tissue of accessory breasts is classified as accessory breast carcinoma. The overall incidence of accessory breast carcinoma is about 0.3%-0.6% (1), with males accounting for only 2.4%-5.3% of these cases (2). Due to the scarcity of documented cases, the management of male accessory breast carcinoma remains nonstandardized, complicating the formulation of evidence-based diagnostic and therapeutic protocols. Here we present a case of male axillary accessory breast carcinoma. Given its exceptional rarity, we herein describe the diagnostic challenges, therapeutic approach, and clinicopathological characteristics to augment the limited clinical experience with this entity.

## 2 Case presentation

A 72-year-old male presented to our clinic with a right axillary mass that had been present for three years. The patient first noticed the mass approximately three years ago, when it measured approximately 0.8×0.5 cm. At that time, the mass was painless and not associated with any redness, swelling, ulceration, or other discomfort. Physical examination at initial discovery showed no changes in the skin or contour of either breast, no nipple discharge or bleeding, and no swelling or pain in the right upper limb or neck region.

Over the following three years, although the patient reported no particular discomfort, he noticed gradual enlargement of the mass. During the most recent 1–2 years, the mass grew to approximately 1.5×0.8 cm, became raised above the skin surface, and developed tenderness when pressed, though it remained without spontaneous pain, local redness, congestion, or ulceration. Repeated examinations confirmed no changes in breast skin or contour, no nipple discharge or bleeding, and no swelling or pain in the right upper limb or neck region.

**Figure 1:**
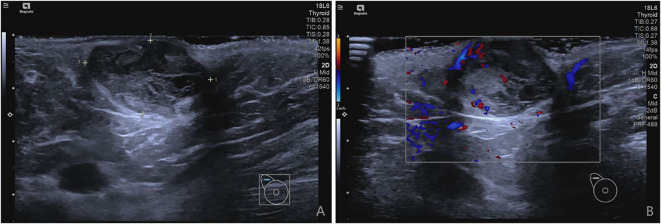
**(A, B)** demonstrate ultrasonographic findings of a solid hypoechoic nodule in the right axilla.

On current physical examination, we identified a well-circumscribed, smooth-surfaced, mobile mass (2.5×1×1 cm) elevated above the skin in the right axilla. The lesion showed no ulceration, vesicle formation, erythema, edema, or *peau d’orange sign*. No vascular bruits were auscultated. Examination of both breasts revealed no abnormalities. Ultrasonography demonstrated a solid hypoechoic mass (29.3 mm × 13.7 mm × 16.2 mm) located 9 mm beneath the epidermal surface in the right axilla. The lesion exhibited well-defined margins, regular morphology, and heterogeneous internal echogenicity. Color Doppler flow imaging (CDFI) revealed moderately abundant intralesional vascularity (**Figures 1A, B**). Preoperative abdominal ultrasonography and chest radiography revealed no detectable abnormalities.

The patient’s medical history was notable for long-term tobacco use (15 years, averaging 10 cigarettes per day) and regular alcohol consumption (10 years, averaging 100 mL per day). No other significant medical history or family history of malignancy was reported.

The patient underwent transaxillary subcutaneous mass excision under local anesthesia. The mass was completely excised. Intraoperative findings revealed a 2.5 cm × 1.0 cm × 1.0 cm cystic-solid mass with a tan-brown surface and a well-circumscribed capsule. Sectioning of the mass demonstrated a cystic component containing colorless, transparent gelatinous fluid, while the solid component exhibited moderate consistency.

**Figure 2:**
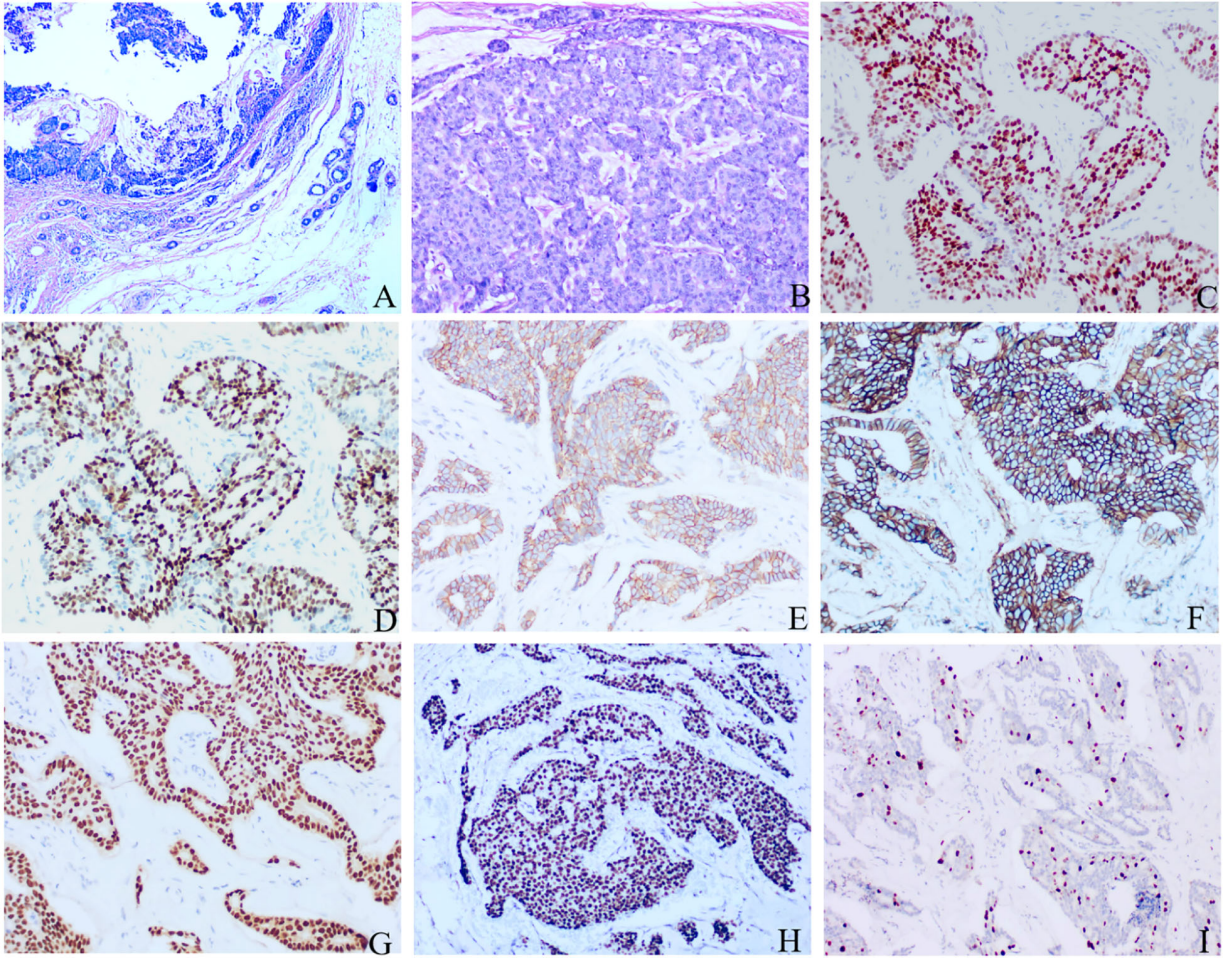
Histopathological and Immunohistochemical of Male Breast Carcinoma **(A)** The tumor is situated within the subcutaneous tissue, unencapsulated, demonstrating infiltrative growth adjacent to cutaneous adnexal eccrine glands. **(B)** Neoplastic cells exhibit solid sheet-like proliferation with uniform cellular size, conspicuous nucleoli, and a mitotic count of 10 per 2 mm². Immunohistochemical Profile: ER (strong nuclear +, 90%) **(C)**, PR (strong nuclear +, 80%) **(D)**, E-cad (+) **(E)**, P120 (membrane +) **(F)**, GATA-3(+) **(G)**, TRPS1(+) **(H)**, Ki-67 (10%+) **(I)**.

Histopathological examination revealed a tumor involved the subcutaneous tissue, without a definitive capsule or lymphoid stroma, with infiltrative growth patterns adjacent to cutaneous adnexal eccrine glands (**Figure 2A**). Neoplastic cells were arranged in tubular and solid sheet patterns, demonstrating moderate cytological atypia characterized by eosinophilic cytoplasm, conspicuous nucleoli, and absence of apocrine snouts. The mitotic count was approximately 10 per 2 mm² (**Figure 2B**). Morphological features were consistent with mammary-origin invasive ductal carcinoma. The resection margins were negative and without lymphovascular invasion.

Immunohistochemistry: CK-Pan (+), CK7 (+), ER (strong nuclear positivity, 90%; **Figure 2C**), PR (strong nuclear positivity, 90%; **Figure 2D**), AR (moderate-to-strong nuclear positivity, 90%), E-cad (+; **Figure 2E**), P120 (membranous +; **Figure 2F**), GATA-3 (+; **Figure 2G**), TRPS1 (+; **Figure 2H**). CK5/6 (−), HER2 (0), P53 (−), CD117 (−), P63 (−), neuroendocrine markers CD56 (−), Syn (−), CgA (−), INSM1 (−). Ki-67 (10% +; **Figure 2I**).

Diagnosis: Invasive ductal carcinoma, not otherwise specified, grade II, right axilla.

Subsequent Therapeutic Interventions.

Based on the histopathological findings, the multidisciplinary tumor board recommended guideline-directed care comprising: I, systemic staging via breast and axillary Magnetic Resonance Imaging (MRI), thoracic/abdominal Computed Tomography (CT) scan; II, additional comprehensive axillary lymph node dissection; III, adjuvant chemotherapy combined with endocrine therapy based on hormone receptor status. The patient declined the recommended interventions. Sutures were removed on postoperative day 10, with the incision achieving Grade I/A healing. At the 11-month follow-up, the right axillary surgical site exhibited optimal wound healing without evidence of palpable lymphadenopathy.

## 3 Discussion

The common risk factors for male breast cancer include: ① age, specifically males aged 60 years and above; ② endocrine factors such as elevated estrogen levels and testicular diseases; ③ obesity and physical inactivity; ④ genetic predisposition in first-degree relatives; and ⑤ radiation exposure.In the current follow-up, the patient was found to have adverse lifestyle habits characterized by a long-term history of tobacco and alcohol use (smoking 10 cigarettes per day for 15 years, and consuming 100 ml of alcohol per day for 10 years). Long-term tobacco and alcohol use is a high-risk factor for tumors. Previous studies have demonstrated that smoking and alcohol consumption increase the risk of breast cancer in females (3, 4); however, there is a lack of research on the correlation with male accessory breast cancer. The report of this case serves as a reminder in this regard.

Accessory breast tissue may occur at diverse anatomical sites, most frequently in the axillary region (approximately 70% of cases), followed by the chest wall, abdominal wall, and inguinal regions (1, 5). The incidence of accessory breast tissue in males is exceedingly low, and clinicians may not readily consider accessory breast pathologies during initial evaluation. This case underscores the necessity of including accessory breast carcinoma in the differential diagnosis when evaluating superficial masses in the axilla, chest wall, abdominal wall, or inguinal regions, even when clinical suspicion is low.

The predominant histological subtype of accessory breast carcinoma is invasive ductal carcinoma, accounting for 70%–90% of reported cases (6, 7). This malignancy is clinically insidious, posing significant diagnostic challenges in early stages. Initial clinical manifestations typically involve painless masses in the axillary or inguinal regions, while ancillary symptoms such as localized swelling, mastalgia, or nipple discharge are less frequently reported (8, 9). The clinical presentation in our reported case aligns with this phenotypic pattern.

The following entities constitute critical differential diagnoses requiring comprehensive evaluation (10–15): ① Accessory breast tissue-related pathologies, (e.g., fibroadenomas, including juvenile gynecomastia in adolescent males); ② Sebaceous cysts; ③ Lipomas; ④ Benign or malignant neoplasms arising from cutaneous adnexal structures; ⑤ Lymphadenopathy, encompassing benign entities (e.g., lymphadenitis, reactive lymphoid hyperplasia, tuberculous lymphadenitis) and malignant conditions (e.g., lymphoma, metastatic carcinoma); ⑥ Hidradenitis suppurativa; ⑦ Castleman disease. (**Table 1**).

**Table 1:** Common disease types and differential diagnosis points of axillary masses.

Disease	Clinical Manifestations	Ancillary Tests	Histopathological
Fibroadenoma	Single or multiple painless masses exhibiting firm consistency, well-defined margins, and high mobility. Characterized by slow growth, with the majority of patients remaining asymptomatic.	Ultrasonography demonstrates a well-circumscribed hypoechoic nodule with homogeneous internal echogenicity and minimal or mild vascularity. Radiographic examination typically reveals no abnormalities.	Histopathological examination demonstrated a tumor composed of mature fibrous and epithelial tissues, with regular cellular morphology and absence of cytological atypia.
Gynecomastia	Breast enlargement, either unilateral or bilateral, is typically characterized by soft consistency and ill-defined margins without palpable discrete masses. Pain and skin adherence are generally absent, though some patients may experience breast tenderness or tenderness on palpation.	Ultrasonography demonstrates diffuse glandular thickening with homogeneous or mildly enhanced echogenicity, without identifiable discrete masses. Mammographic evaluation typically reveals no abnormalities unless concurrent breast pathology is present.	Ductal and lobular hyperplasia with stromal edema, absence of malignant cells.
Sebaceous Cyst	Sebaceous cysts primarily occur in sebaceous gland-rich regions, such as the head, face, back, and buttocks, and are typically absent in accessory breast regions. These lesions present as rounded masses with moderate consistency, well-defined margins, and high mobility. A central punctum (a dark, pinpoint-sized depression) is frequently observed at the lesion’s apex. Pain is uncommon unless secondary infection occurs, which may manifest as erythema, localized tenderness, and purulent discharge.	Ultrasonography typically demonstrates a well-circumscribed cystic lesion with homogeneous internal echogenicity and no significant vascularity.	Histopathological examination reveals a cyst wall lined by stratified squamous epithelium, with the lumen filled with keratinous material and sebum.
Lipoma	Lipomas may arise in any adipose tissue-containing regions of the body, most commonly within subcutaneous tissue such as the shoulders, back, and abdomen. These lesions typically present as solitary or multiple round or lobulated masses characterized by soft consistency, well-defined margins, and high mobility. Growth is slow, and the majority of cases remain asymptomatic.	Ultrasonography typically demonstrates a well-circumscribed hyperechoic or isoechoic mass with homogeneous internal echogenicity, posterior acoustic enhancement, and minimal or absent vascularity.	Histopathological examination reveals mature adipocytes encapsulated within an intact fibrous capsule, demonstrating normal cellular morphology and absence of significant cytological atypia.
Benign and Malignant Tumors of Cutaneous Adnexa	Lesions predominantly involve the cutaneous layer, with potential extension into subcutaneous tissue, and are most frequently observed in the head, facial, and cervical regions—distinct from the anatomical distribution of accessory breast tissue. Surface manifestations may include erosion, ulceration, or hemorrhage. Owing to firm adhesion between the mass and adjacent cutaneous/subcutaneous structures, mobility is significantly restricted. Some patients exhibit localized symptoms such as pruritus or tenderness, accompanied by cutaneous hyperpigmentation or hypopigmentation.	Benign lesions typically exhibit well-circumscribed margins with a regular round or oval configuration, demonstrating clear demarcation from surrounding tissues. Internal echogenicity is predominantly homogeneous, though its intensity may vary depending on the specific lesion type. Exemplar case: Sebaceous cysts often present as anechoic or hypoechoic lesions containing internal hyperechoic foci, typically accompanied by minimal or absent vascularity. Malignant lesions are characterized by ill-defined margins, irregular morphology (e.g., lobulated or spiculated contours), and infiltrative growth into adjacent tissues. Ultrasonography demonstrates heterogeneous internal echogenicity, often with complex architectural features such as calcifications and cystic components. Doppler imaging typically reveals hypervascularity with elevated flow velocity and low resistance index.	Histopathological examination revealed neoplastic tissue exhibiting glandular, ductal, papillary, diffuse, or solid growth patterns, predominantly localized to the deep dermis with frequent extension into the subcutaneous adipose tissue. The epithelial cells demonstrated abundant eosinophilic cytoplasm and distinct apocrine differentiation (e.g., apical snouts).
Lymph Node	Enlargement of axillary lymph nodes may present as a localized mass. Affected lymph nodes may exhibit variable consistency (firm to soft), relatively well-defined margins, and preserved mobility. Inflammatory lymphadenopathy is typically associated with tenderness and erythema, whereas metastatic lymphadenopathy secondary to tumor dissemination often demonstrates an absence of significant pain.	Normal lymph nodes exhibit an oval shape with distinct corticomedullary differentiation and minimal vascularity. In contrast, enlarged lymph nodes may demonstrate irregular morphology, cortical thickening, and increased vascularity. Contrast-enhanced CT or PET-CT imaging is critical for evaluating metastatic lymphadenopathy and assessing systemic involvement.	Histopathological examination can determine the etiology of lymphadenopathy, including inflammatory processes, tuberculosis, or metastatic involvement. In cases of metastatic disease, it may further localize the primary tumor site.
Hidradenitis suppurativa	A chronic and recurrent skin disease characterized by suppurative inflammation. It typically occurs in areas where apocrine glands are distributed, such as the axillae and groin. In the early stage of the disease, it manifests as small red nodules, which subsequently progress into abscesses. Once ruptured, purulent secretions are discharged, and sinus tracts and fistulas may form, with recurrent episodes. Generally, there is no obvious pain. The skin may show redness, swelling, and ulceration, but typical symptoms of breast cancer, such as peau d’orange changes or dimpling signs, will not be present.	Ultrasonography demonstrates localized inflammatory changes.	Histopathological examination reveals inflammatory changes in sweat glands and abscess formation, with no evidence of malignant cells.
Castleman disease	Castleman disease is postulated to arise from chronic inflammatory stimulation driving abnormal lymphoid hyperplasia. Clinical manifestations vary significantly depending on the disease subtype: 1.Focal type: Typically presents as solitary or regional lymphadenopathy characterized by firm consistency and painless progression. 2.Multicentric type: Manifests with systemic B symptoms including fever, fatigue, night sweats, and weight loss; Accompanied by generalized lymphadenopathy and hepatosplenomegaly in advanced stages.	Ultrasonography may demonstrate lymphadenopathy with diverse morphological features, exhibiting homogeneous or heterogeneous internal echogenicity. Contrast-enhanced CT or magnetic resonance imaging (MRI) can better delineate the lesion extent and its anatomical relationship with adjacent structures. In Castleman disease, affected lymph nodes typically exhibit hypervascularity and marked enhancement on contrast imaging.	Histopathological examination reveals lymphoid follicular hyperplasia within the affected lymph nodes, characterized by prominent germinal centers and interfollicular vascular proliferation with dense plasma cell infiltration. Based on histomorphology features, Castleman disease is classified into three subtypes: Hyaline vascular type、Plasma cell type、Mixed type. Immunohistochemical analysis demonstrates positive expression of follicular dendritic cell markers (e.g., CD21, CD23), along with variable expression of B-cell markers (e.g., CD20, PAX5) and T-cell markers (e.g., CD3, CD5).

For axillary masses with postoperative pathological confirmation of breast carcinoma, the diagnostic workup must first exclude the following possibilities: ① Secondary metastatic carcinoma, particularly axillary lymph node metastases from primary adenocarcinomas of the breast, gastrointestinal tract, prostate, or lung that may mimic subcutaneous axillary masses; ② For pathological diagnosis, the pathological type should support that the heteromorphic cells originate from the mammary glands. Since the mammary glands, sebaceous glands, and sweat glands all originate from the same stem cells, during the diagnostic process, immunohistochemical auxiliary diagnosis should be employed to ensure the accuracy of the diagnosis.

The histological features of this case necessitate primary differentiation from apocrine carcinoma, a rare malignancy predominantly localized to the axillary region. Apocrine carcinoma typically manifests as solitary or multiple slow-growing nodules or plaques with erythematous to violaceous surfaces, occasionally demonstrating ulceration. Histopathologically, it is characterized by glandular, ductal, papillary, or solid growth patterns predominantly involving the deep dermis and subcutaneous adipose tissue. Tumor cells exhibit abundant eosinophilic cytoplasm with definitive apocrine secretion. The nuclei are round to oval, vesicular, and exhibit a single prominent nucleolus (16, 17).

In contrast, the present case demonstrated tubular and solid growth architectures without papillary or ductopapillary structures. Neoplastic cells displayed moderate eosinophilic cytoplasm devoid of apocrine differentiation, a critical morphological distinction. Immunohistochemically, both entities share overlapping profiles (AE1/AE3, ER/PR/AR, GATA3), limiting their discriminative utility. However, TRPS1—a highly sensitive and specific biomarker for mammary-origin malignancies (18)—exhibited strong positivity in this case, further supporting the diagnosis of accessory breast carcinoma. In summary, histomorphology characteristics, particularly the presence or absence of apocrine differentiation, constitute pivotal diagnostic discriminators between these entities.

The current case was diagnosed as invasive ductal carcinoma. To better understand the pathological features of invasive breast cancer, we also reviewed and compared it with non-invasive carcinoma. The fundamental pathological distinction between invasive and non-invasive carcinoma lies in the integrity of the basement membrane. In non-invasive ductal carcinoma, tumor cells are confined within the ducts without breaching the basement membrane, while in invasive carcinoma, tumor cells penetrate the basement membrane and infiltrate into the stroma or surrounding tissues. Non-invasive carcinomas include ductal carcinoma *in situ* (DCIS), where tumor cells remain within the ductal basement membrane, and lobular carcinoma *in situ* (LCIS), where tumor cells do not breach the basement membrane of terminal ducts or acini. Currently, DCIS is primarily graded based on nuclear features (19) (**Table 2**). LCIS represents an important special lesion that requires differentiation from DCIS. LCIS cells demonstrate poor adhesion and distend ≥50% of acini in the terminal duct-lobular unit, and can be classified as classic, florid, or pleomorphic types. When involving <50% of acini, it is diagnosed as atypical lobular hyperplasia (ALH). Immunohistochemically, LCIS typically shows loss of E-cadherin membranous staining or other abnormal expression patterns, strong diffuse cytoplasmic staining of p120 and β-catenin, and loss of β-catenin membranous staining. The differential diagnosis between LCIS and DCIS should integrate both histomorphology and immunohistochemical markers. Classic and florid LCIS usually show diffuse and homogeneous ER positivity with low Ki-67 index, while the pleomorphic subtype is often ER-negative but may express androgen receptor (AR). HER2 overexpression and gene amplification can also occur, with approximately 10% of pleomorphic LCIS cases being triple-negative (20–22).

**Table 2:** Histopathological features of ductal carcinoma *in situ* (DCIS) by nuclear grade.

Item	Low Nuclear Grade	Intermediate Nuclear Grade	High Nuclear Grade
Cytomorphologic Features	Minimal cellular atypia with uniform nuclear morphology; nuclear size 1.5–2.0× that of red blood cells or normal ductal epithelial cells; hyperchromatic nuclei with evenly distributed chromatin, inconspicuous nucleoli, and pale or eosinophilic cytoplasm	Moderate atypia, intermediate between low and high nuclear grade; granular or clumped chromatin, occasionally visible nucleoli	Marked cellular atypia with pleomorphic nuclei; nuclear size ≥2.5× that of red blood cells or normal ductal epithelial cells; hyperchromatic or vesicular nuclei with irregular contours, coarse chromatin, and prominent nucleoli
Mitotic Figures	Rare or absent	Present	Frequent
Necrosis (including comedonecrosis)	Rare	Focal	Common
Calcification	Uncommon	Present	Common
Immunohistochemistry	Estrogen receptor (ER) and progesterone receptor (PR) typically diffusely strongly positive; HER2 and cytokeratin (CK)5/6 negative; low Ki-67 proliferation index	Variable ER, PR, and HER2 expression; CK5/6 negative; intermediate Ki-67 proliferation index	ER and PR often negative; HER2 frequently positive or triple-negative; CK5/6 may be positive; high Ki-67 proliferation index

Given the limited clinical experience among practitioners, initial diagnostic evaluations are typically restricted to conventional imaging modalities such as Doppler ultrasonography. Advanced modalities including CT, MRI, and positron emission tomography - computed tomography (PET-CT), while diagnostically valuable, are reserved for selective use in clinical practice due to cost and accessibility constraints. For cases with high clinical suspicion of accessory breast carcinoma, image-guided core needle biopsy may be considered (23, 24).

In cases where malignancy demonstrates low clinical suspicion, local excision is usually performed during the index procedure, leading to a definitive diagnosis of accessory breast carcinoma established only after comprehensive postoperative histopathological evaluation. Due to limited evidence, the prognostic implications of this conservative surgical approach remain inconclusive.

Currently, no established therapeutic consensus exists for male accessory breast carcinoma, and clinical management primarily aligns with protocols for male breast cancer (25, 26). Treatment strategies should be tailored according to tumor stage and molecular subtype. Notably, limited evidence supports the routine use of neoadjuvant therapy in male breast cancer, with controversial indications largely extrapolated from female breast cancer guidelines (25). Consequently, neoadjuvant therapy is not recommended as first-line intervention for male accessory breast carcinoma. Surgical resection, including wide local excision with lymph node dissection, remains the cornerstone of definitive management. The performance of a mastectomy depends on anatomical proximity. Modified radical mastectomy should be considered when the lesion is contiguous with native breast tissue. Conversely, preservation of native breast parenchyma is advised if the accessory breast constitutes a separate anatomical entity, as prophylactic mastectomy offers no oncologic benefit. Postoperative adjuvant therapies—including chemotherapy, radiotherapy, endocrine therapy, and targeted therapies—should be individualized based on clinicopathological risk stratification (19, 20). Over 90% of male breast cancers are hormone receptor-positive. Current international guidelines recommend a 5-year tamoxifen regimen, with extended adjuvant therapy (additional 5 years) reserved for high-risk patients demonstrating adequate tolerance (26).

Therefore, if the patient agrees to further treatment, given that this case is ER/PR-positive, HER2-negative, and has a Ki-67 ≤20%—consistent with Luminal A breast cancer—the tumor is likely highly sensitive to endocrine therapy but generally less responsive to chemotherapy. We recommend completing 5 years of tamoxifen therapy. Although some studies have explored neoadjuvant chemotherapy (NAC) in male breast cancer (27), the evidence remains limited, and clinical practice largely extrapolates from female breast cancer data, which remains controversial. Notably, studies report that pathologic complete response (pCR) rates in men are consistently lower than in women, particularly in hormone receptor-positive subtypes (28). Currently, there is insufficient high-quality evidence to guide chemotherapy decisions for male breast cancer patients. Based on available clinical data, TC (docetaxel + cyclophosphamide) or AC (doxorubicin + cyclophosphamide) regimens may be considered for this patient if chemotherapy is pursued.

## 4 Conclusion

Male accessory breast carcinoma is extremely rare, exhibits relatively high malignancy, and has a low 5-year survival rate. Preoperative diagnosis remains one of the most significant challenges. Clinically, when encountering masses in the axillary or inguinal regions, the possibility of accessory breast carcinoma should not be easily excluded regardless of the patient’s sex. Preoperative evaluations should include necessary examinations, and core needle biopsy is a practical diagnostic approach for suspicious lesions. Surgical intervention primarily involves local excision combined with axillary lymph node dissection. Postoperative adjuvant therapies, including chemotherapy, radiotherapy, endocrine therapy, and targeted therapy, should be administered based on individualized assessments.

## Data Availability

The original contributions presented in the study are included in the article/supplementary material. Further inquiries can be directed to the corresponding authors.
