# Correction: Male accessory breast carcinoma: a rare case report and literature review

**DOI:** 10.3389/fonc.2025.1717570

**Published:** 2025-10-15

**Authors:** Cheng Luo, Songli Wu, Ying Zeng, Song Zhao

**Affiliations:** 1 Department of General Surgery, The Thirteenth People’s Hospital of Chongqing (Chongqing Geriatrics Hospital), Chongqing, China; 2 Department of Pathology, The Thirteenth People’s Hospital of Chongqing (Chongqing Geriatrics Hospital), Chongqing, China

**Keywords:** accessory breast carcinoma, male breast cancer, axillary mass, TRPS1, immunohistochemistry

The order of authors in the author list of the published paper was erroneously given as:

“Cheng Luo, Songli Wu, Song Zhao and Ying Zeng”

The correct author list reads:

“Cheng Luo, Songli Wu, Ying Zeng and Song Zhao”

The **Author contributions** statement was erroneously given as “CL: Writing – original draft. SW: Writing – original draft, Writing – review & editing. SZ: Writing – review & editing, Supervision. YZ: Writing –original draft”. The correct **Author contributions** statement is “CL: Writing – original draft. SW: Writing – original draft, Writing – review & editing. YZ: Writing – original draft. SZ: Writing – review & editing, Supervision”.

Author “CL and SW contributed equally to this work and share first authorship” was erroneously omitted as equal contributing author.

The original version of this article has been updated.

